# Oskar Frischenschlager, Birgit Hladschik-Kermer (Hrsg.): Gesprächsführung in der Medizin – lernen, lehren, prüfen

**DOI:** 10.3205/zma000957

**Published:** 2015-05-13

**Authors:** Anne Herrmann-Werner

**Affiliations:** 1 Universitätsklinikum Tübingen, Innere Medizin VI, Abteilung Psychosomatische Medizin und Psychotherapie, Tübingen, Deutschland; 2 Eberhard-Karls-Universität Tübingen Medizinische Fakultät, Leiterin des Tübinger Interdisziplinären Skills Labs (DocLab), Tübingen, Deutschland

## Recension

The issue of communication in the doctor-patient-relationship is of uttermost importance and has additionally gained a central position in medical education ever since its importance has been stressed through the novellation of the German Medical Licensure Act in 2012 as well as within the German National Catalogue of Learning Objectives. It is commonly accepted that communication has to be taught and learned – as opposed to the earlier view of it being acquired as just a sideline. 

The book “Gesprächsführung in der Medizin – lernen, lehren, prüfen” addresses the issue from different perspectives. In the first chapter, basic concepts of communication are graphically introduced. Desirably, aspects from other fields related to communication (e.g. communication studies, psychology) would have been presented as well – alongside with well-known models (like Schulz von Thun or Watzlawick).

In the second part of the book, communicational competencies are introduced and discussed. This gives the reader a very good basis and additionally ideas how doctors themselves could profit from successful communication or seek support in difficult situations as needed, respectively. 

The following segment introduces three selected special situations of communication (palliative patient, ward round, children). They give a good first impression though they are surely not entirely comprehensive. 

The forth chapter is probably the most interesting one, as it deals with the actual teaching of communicational competencies. It describes aspects of different settings (one-to-one, team) as well as various possibilities of examinations and the use of standardized patients. Positively surprising is the inclusion of the question which qualification is required of teachers as well as the compilation of a toolbox with established models (e.g. SPIKES, NURSE). 

The final part of the book introduces the longitudinal curriculum in Vienna as an example for successful implementation. 

In summary, this book gives a basic overview on the topic of doctor-patient-communication. Pretty helpful are the practical case examples with clinical orientation, which could have been a bit more numerous. This also shows the book’s general heterogeneity: depending on the respective author, the chapters are written more or less clearly as well as have been checked for redundancies. 

All in all, it can be stated that if you have already dealt with the issue of teaching communication in the medical field, there won’t be much new information in the book. But if you are looking for a good introduction to this topic, the Frischenschlager/Hladschik-Kermer is definitely a good choice for you. 

## Competing interests

The author declares that she has no competing interests. 

